# Comparing the predicted accuracy of PO_2_\FIO_2_ ratio with rapid shallow breathing index for successful spontaneous breathing trial in Intensive Care Unit

**DOI:** 10.12669/pjms.35.6.788

**Published:** 2019

**Authors:** Aamir Furqan, Shumaila Ali Rai, Liaqat Ali, Rana Altaf Ahmed

**Affiliations:** 1 Dr. Aamir Furqan, MBBS, FCPS (Anaesthesia), FCPS (Cardiothoracic Anaesthesia). Department of Anaesthesia and ICU, Ch. Pervaiz Ellahi Institute of Cardiology, Multan, Pakistan; 2 Dr. Shumaila Ali Rai, MBBS, FCPS. Department of Anaesthesia and ICU, Ch. Pervaiz Ellahi Institute of Cardiology, Multan, Pakistan; 3 Dr. Liquat Ali, MBBS, FCPS. Department of Anaesthesia and ICU, Multan Medical and Dental College, Multan, Pakistan; 4 Dr. Rana Altaf Ahmed, MBBS, FCPS. Department of Anaesthesia and ICU, Ch. Pervaiz Ellahi Institute of Cardiology, Multan, Pakistan

**Keywords:** PO_2_\FIO_2_ ratio, Predictive value, Rapid shallow breathing index, Sensitivity, Specificity, Spontaneous breathing trial

## Abstract

**Objective:**

To compare the predicted accuracy of PFR with RSBI for successful spontaneous breathing trial before extubation in intensive care unit.

**Methods:**

This cross sectional study was conducted at the ICU of Ch. Pervaiz Ellahi Institute of Cardiology, Multan Medical and Nishtar Medical University Hospital from July, 2017 to January, 2019.PO_2_/FIO_2_ and RSBI was measured by a different investigator, before and 20 minutes after the start of SBT. Heart rate, blood pressure and oxygen saturation were continuously measured throughout the trial. Trial outcome was labeled as unsuccessful or successful by the investigator who was blinded to the rapid shallow breathing index and PO2/FiO2 measurements. Patients with SpO2>85%, stable hemodynamics (HR and BP change <20%), stable respiration (RR change <50%), and the absence of (i) signs of labored breathing, (ii) emergence or worsened discomfort, (iii) change in mental status, were labeled as successful in bearing the SBT. Patients were divided into two groups i.e. successful and unsuccessful, gender, Age, GOLD stage, APACHE II score, pCO2, pO2, FiO2 and RSBI score were compared between the two groups after putting all the data in SPSS version 23. Chi square tests and Student’s t-test were used on the continuous data and nominal data, accordingly. The specificity, sensitivity, diagnostic accuracy, negative predictive value and positive predictive value of two threshold values of RSBI and PO2/FiO2 ratio were calculated from the 2X2 contingency tables.

**Results:**

RSBI threshold of 130 had 40.4% sensitivity, 51.1% specificity, 55.2% positive predictive value, 36.4% negative predictive value and 44.7% diagnostic accuracy while RSBI threshold of 105 had 94.1% sensitivity, 43.6% specificity, 71.4% positive predictive value, 83.2% negative predictive value and 73.8% diagnostic accuracy. pO_2_/FiO_2_>250 had 76.9% sensitivity, 24.5% specificity, 60.4% positive predictive value, 41.5% negative predictive value and 55.9% diagnostic accuracy.

**Conclusion:**

Even though neither rapid shallow breathing nor the PFR was enough accurate in prediction of successful extubation but rapid shallow breathing index 105 threshold had higher sensitivity and specificity as compared to RSBI threshold 130PFR. Therefore, RSBI105 is more accurate in predicting the outcome of extubation of ICU patients.

## INTRODUCTION

Detection of the patient who is able to breathe simultaneously is an important aspect of management of the patients in ICU.^1^ There are multiple criteria which are used to predict whether a patient is breathing spontaneously. Studies have shown that no single predictor is effective enough in predicting the spontaneous breathing in ICU patients.^2^ Ratio of tidal volume and respiratory rate is termed as rapid shallow breathing index and has been widely used in previous studies in prediction of outcome of spontaneous breathing trial.^3^ According to the reports almost 40 percent of the patients in ICU suffering from pulmonary diseases require mechanical ventilation.^4^ Extubation in most of the patients is performed at 2 to 4^th^ day after ventilation and in round about 25% of the patients the mechanical ventilation continues even after seven days of intubation.^5^ There are number of weaning protocols, daily based spontaneous breathing trials and automated systems which are followed in ICU during extubation of the patients but survey suggests that among 20 to 30 percent of the patients, first attempt at extubation is failed.^6^

Rapid shallow breathing index and spontaneous breathing trials have been used as the predictors of thriving weaning from mechanical ventilation.^6^ However, their imperfection compels the physicians to include other clinical criteria into consideration for the prediction of successful extubation. Clinical judgment is sole traditional way of making the decision of extubation. These clinical judgments are based upon following parameters e.g. continuous positive airway pressure tolerated at 5-7 cmH_2_O without any signs of fatigue for twelve hours, arterial PO_2_>80mmHg at room air and bulbar paresis improved etc. Among the different modalities used for the prediction of outcome of extubation in ICU patient, diaphragm ultrasound has also been used in mechanically ventilated patients.^7^ Similarly different spontaneous breathing trials have also been studied in past literature in prediction of failure rate of extubation.

Ratio PaO_2_/FiO_2_ is the ratio of partial pressure of arterial oxygen to the fractional inspired oxygen. It is also known as Carrico index or PF ratio. It is most commonly used as a clinical indicator of level of hypoxemia but its diagnostic accuracy is doubtful. In previous literature PaO_2_/FiO_2_ ratio has been implemented and studied as a predictor of outcome of extubation in patients with mechanical ventilation but most of the studies have deduced that it is not a reliable predictor.^8^ However most of these studies have been carried out on the patients suffering from hypercapnic respiratory failure. Precision of all the methods mentioned above is not completely satisfactory. Therefore, studies are needed to determine a more accurate way of prediction of success of extubation among the patients with mechanical ventilation in intensive care units. Not much work in this regard has been done in previous studies, so the need to conduct this study in which Comparison between the predicted accuracy of PO_2_\FIO_2_ ratio with rapid shallow breathing index for successful spontaneous breathing trial was conducted. The observations of our study is helpful to adopt more sensitive indicator of extubation.

## METHODS

This is a cross sectional descriptive study which was conducted on the patients who were admitted in the ICU of Nishtar Medical University Hospital and Ch. Pervaiz Ellahi Institute of Cardiology, Multan. We included 1500 patients who were admitted from July, 2017 to January, 2019. All the included patients were ready to undergo spontaneous breathing trial. Before starting the study, ethical approval was obtained from the hospital review board. Written consent was obtained from the first degree relatives of all the patients included in study. Patients who are clinically stable and have the criteria for weaning from the ventilator, patients who are intubated for at least 48 hours were included in the study. Patients who are clinically unstable, evidence of myocardial ischemia, heart rate (HR) >140 beats/min, patients with fever and significant electrolyte abnormalities, high vasopressor requirement (i.e., >5 mcg/min of noradrenaline) for maintaining blood pressure were excluded.

Weaning criteria was respiratory rate less than 25 breaths per minute, tidal volume greater than 5 mL/kg, vital capacity greater than 10 mL/k, minute ventilation less than 10 L/min, PaO_2_/FIO_2_ greater than 200, Shunt (Qs/Qt) less than 20%, negative inspiratory force (NIF) less than (more negative) -25 cm water, f/Vt less than 105, or less than 130 in elderly patients. Age, gender, pulmonary function tests, APACHE II score, days on mechanical ventilation, FiO_2,_ hemoglobin, serum sodium, potassium and calcium were recorded before the start of the spontaneous breathing trial.

Arterial blood gas analysis was done before and after the commencement of SBT. RSBI was defined as the ratio of breathing rate to the tidal volume per liter. It was measured after five minutes of the start of SBT. A good indicator of enduring SBT is the pattern of breathing during first 3-5 minutes.^9^ Measurements within first 5 minutes and calculated the diagnostic accuracy of the RSBI in predicting the outcome of the weaning trial. After disconnecting the mechanical ventilation, a handheld Wright spirometer was placed in front of the endotracheal or tracheostomy tube of the patients. The pulmonologist calculated the respiratory rate. Minute ventilation was divided by the rate of respiration for tidal volume calculation.^10^ Two threshold values i.e. 105 bmp/L and 130bp2m/L were evaluated. Yang’s study^11^ and Vallverdu et al.^12^ also used same criteria.

Patients were put on T-piece after thorough suctioning. The oxygen flow was set to arterial oxygen saturation above 90%. Heart rate, blood pressure and oxygen saturation were continuously measured throughout the trial. Trial outcome was labeled as unsuccessful or successful by the investigator who was blinded to the rapid shallow breathing index and PO2/FiO2 measurements. Patients with SpO2>85%, stable hemodynamics (HR and BP change <20%), stable respiration (RR change <50%), and the absence of (i) signs of labored breathing, (ii) emergence or worsened discomfort, (iii) change in mental status, were labeled as successful in bearing the SPT. Patients were divided into two groups i.e. successful and unsuccessful. Patients who were not extubated gently deterioration of vitals and saturation or need intubation again was labeled as unsuccessful. Gender, Age, GOLD stage, APACHE II score, pCO2, pO2, FiO2 and RSBI score were compared between the two groups after putting all the data in SPSS version 23. Chi square tests and Student’s t-test were used on the continuous data and nominal data, accordingly. The specificity, sensitivity, diagnostic accuracy, negative predictive value and positive predictive value of two threshold values of RSBI and PO2/FiO2ratio were calculated from the 2X2 contingency tables.

**Fig. 1:**
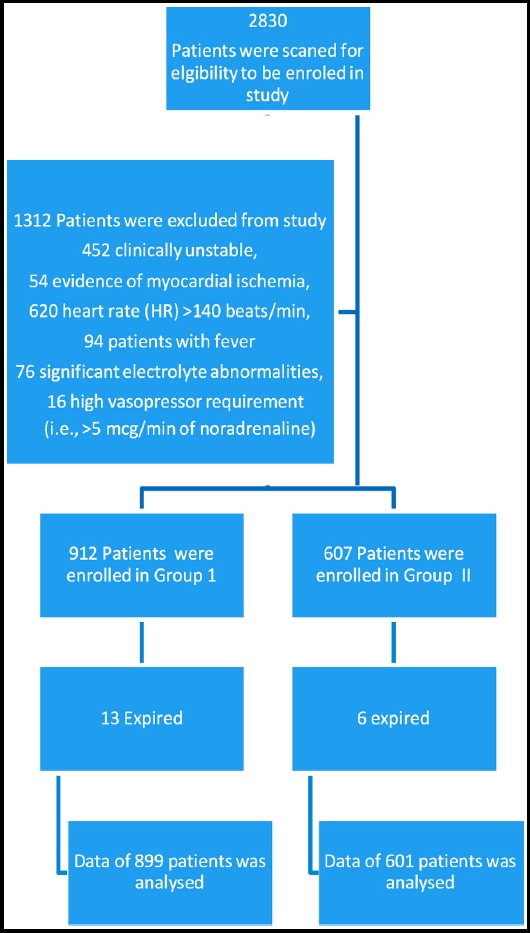
Flow Sheet of Study Patients.

## RESULTS

Of all the patients, 899 patients completed spontaneous breathing trial without clinical deterioration, while 601 patients did not remain clinically stable and were returned to the mechanical ventilation. No significant difference of age, gender distribution, GOLD stage, pO2, pCO2 and FiO2 were observed between the two groups. There were 723 males and 176 females in Group-1 while 466 males and 135 females in Group-2. Group-1 patients stayed for 6.34 ± 2.45 days on mechanical ventilation and had 14.48 ± 2 APACHE II score while Group-2 patients had 6.50 ± 2.01 days stay on mechanical ventilation and had 14.32 ± 2 APACHE II score (0.182 and 0.125, respectively). RSBI score was 109.28±23.26 in Group-1 and 115.42±31.16 in group-2 and the difference was of statistical significance (p<0.001) Table-I.

**Table I:** Basic characteristics.

Variable	Group-1 (n=899)	Group-2 (n=601)	p-value
Age, years	42.34±15.52	43.57±8.76	0.052
Gender (male/female)	723/176	466/135	0.177
GOLD stage, N (%)			
II	104 (11.6)	80 (13.3)	0.172
III	246 (27.4)	140 (23.3)	
IV	549 (61.1)	381 (63.4)	
Days on mechanical ventilation	6.34±2.45	6.50±2.01	0.182
APACHE II score	14.48±2	14.32±2	0.125
pO_2_	97.85±9.79	98.97±12.12	0.059
pCO_2_	47.57±7.29	47.24±6.53	0.368
RSBI score	109.28±23.26	115.42±31.16	<0.001
FiO_2_	33.49±8.77	34.07±8.98	0.217

At 105 threshold value of RSBI, 657 patients were expected to complete spontaneous breathing trial and only 363 completed while 843 patients were expected to fail of which 536 completed trial (Table-II). At 130 threshold value of RSBI, 1185 patients were expected to complete spontaneous breathing trial and only 846 completed while 315 patients were expected to fail of which 53 completed trial (Table-III). At pO_2_/FiO_2_ >250, 1146 patients were expected to complete spontaneous breathing trial and only 692 completed while 354 patients were expected to fail of which 207 completed trial (Table-IV).

**Table II:** 2X2 table for RSBI <130.

		Patients outcome		Total

		Successful	Unsuccessful	
Expected outcome	Successful	363	294	657
	Unsuccessful	536	307	843

Total		899	601	1500

**Table III:** 2X2 table for RSBI <105.

		Patients outcome		Total

		Successful	Unsuccessful	
Expected outcome	Successful	846	339	1185
	Unsuccessful	53	262	315

Total		899	601	1500

**Table IV:** 2X2 table for pO_2_/FiO_2_>250.

		Patients outcome		Total

		Successful	Unsuccessful	
Expected outcome	Successful	692	454	1146
	Unsuccessful	207	147	354

Total		899	601	1500

RSBI threshold of 130 had 40.4% sensitivity, 51.1% specificity, 55.2% positive predictive value, 36.4% negative predictive value and 44.7% diagnostic accuracy while RSBI threshold of 105 had 94.1% sensitivity, 43.6% specificity, 71.4% positive predictive value, 83.2% negative predictive value and 73.8% diagnostic accuracy. pO_2_/FiO_2_>250had 76.9% sensitivity, 24.5% specificity, 60.4% positive predictive value, 41.5% negative predictive value and 55.9% diagnostic accuracy Table-V.

**Table V:** Sensitivity, specificity, negative and positive predictive values, and diagnostic accuracy for two threshold values of RSBI and pO_2_/FiO_2_>250.

Variable	RSBI≤130	RSBI≤105	pO_2_/FiO_2_>250
Sensitivity	40.4%	94.1%	76.9%
Specificity	51.1%	43.6%	24.5%
Positive predictive value	55.2%	71.4%	60.4%
Negative predictive value	36.4%	83.2%	41.5%
Accuracy	44.7%	73.8%	55.9%

## DISCUSSION

Rapid shallow breathing index less than 130 had 40.4% sensitivity while its specificity was 51.1%. Important factors resulting in the failure of the extubation have been described by Krieger et al.^13^ and Miu et al.^14^ One of these factors was oxygenation which is an important factor in early failure of extubation. Moreover, continuous and repeated failure of spontaneous breathing trials and decreased levels of diastolic blood pressure also contribute to the failure of extubation at any time.

In a nstudy rapid shallow breathing index was used in the prediction of the requirement of noninvasive ventilation among sixty-one patients.^15^ Rapid shallow breathing index helped in prediction of the need of the noninvasive ventilation in 26 of the patients and 35 of the patients did not need noninvasive ventilatory support. Mean rapid shallow breathing index among the patients who did not require noninvasive ventilation was 130 (p=0.0001). Sensitivity and specificity of rapid shallow breathing index >120 was 81% and 74% respectively for determination of the requirement of the noninvasive ventilation. These findings are different from the findings of our study where rapid shallow breathing index of less than 120 had a sensitivity and specificity of 94.1% and 43.6% respectively.

Similarly, in another study requirement of noninvasive ventilation was predicted using rapid shallow breathing index and APACHE II score.^16^ Need of noninvasive ventilation was seen in 43.9% of the patients. Sensitivity and specificity of rapid shallow breathing index and APACHE score was 94.8% and 72% respectively which is comparable to the results of our study. Moreover, no ABG parameters such as PO2 or FIO2 were significant in determining the requirement of noninvasive ventilatory support among the patients of chronic obstructive pulmonary disease.

In another study 191 patients were studied and rapid shallow breathing index was applied in spontaneous breathing trial and it was found that weaning from intubation was successful in 165 of the patients while failed in 26 of them. These results are in favor of the use of rapid shallow breathing index in predicting the weaning outcome.^17^

Very few studies have been seen in which PO2:FIO2 ratio was used as a prediction tool for the outcomes assessment among ICU patients before extubation. In a previous study predictive value of PFR (PO2:FIO2 ratio) was assessed both with standardized rapid shallow breathing index and independently for successful extubation in patients with hypoxemic respiratory failure. The outcome against which the efficacy of PFR was measured was the requirement of reintubation within 48 hours of extubation. The results of that study showed that 92% were successfully extubated. The value of PFR and RSBI (rapid shallow breathing index) among the patients who were successfully extubated and those who required reintubation were almost similar. A PFR of >200 and RSBI >70 with PFR>200 were present the risk of reintubation was higher with sensitivity and specificity of 70% and 56% respectively. Conclusion on the basis of these results was made that PFR is not useful technique for the prediction of successful extubation whether used alone or with RSBI in patients with hypoxemic respiratory failure.^18^

In yet another study where different factors were studied for the prediction if successful extubation the PFR was found significant in prediction of death in bivariate analysis while it was not statistically significant in prediction among multivariate adjusted analysis.^19^

## CONCLUSION

Results of this study shows that neither rapid shallow breathing nor the PFR was accurate in prediction of successful extubation but rapid shallow breathing index had higher sensitivity and specificity as compared to PFR. Therefore, RSBI is more accurate in predicting the outcome of extubation of ICU patients.

### Author`s Contribution:

**AF** conceived, designed and did statistical analysis & editing of manuscript. Is responsible for integrity of research.

**SAR and LA** did data collection and manuscript writing.

**RAA** did review and final approval of manuscript.

## References

[1] Yoshida T, Fujino Y, Amato MB, Kavanagh BP (2017). Fifty years of research in ARDS. Spontaneous breathing during mechanical ventilation. Risks, mechanisms, and management. Am J Resp Critic Care Med.

[2] Teixeira SN, Osaku EF, de Macedo Costa CR, Toccolini BF, Costa NL, Candia MF (2015). Comparison of proportional assist ventilation plus, T-tube ventilation, and pressure support ventilation as spontaneous breathing trials for extubation:a randomized study. Respir Care.

[3] Karthika M, Al Enezi FA, Pillai LV, Arabi YM (2016). Rapid shallow breathing index. Ann Thorac Med.

[4] Del Sorbo L, Goligher EC, McAuley DF, Rubenfeld GD, Brochard LJ, Gattinoni L (2017). Mechanical ventilation in adults with acute respiratory distress syndrome. Summary of the experimental evidence for the clinical
practice guideline. Ann Am Thorac Socie.

[5] Perkins GD, Mistry D, Gates S, Gao F, Snelson C, Hart N (2018). Effect of Protocolized Weaning with Early Extubation to Noninvasive Ventilation vs Invasive Weaning on Time to Liberation From Mechanical Ventilation Among Patients With Respiratory Failure:The Breathe Randomized Clinical Trial. JAMA.

[6] Abbas A, Embarak S, Walaa M, Lutfy SM (2018). Role of diaphragmatic rapid shallow breathing index in predicting weaning outcome in patients with acute exacerbation of COPD. Int J Chronic Obstr Pulmon Dis.

[7] DiNino E, Gartman EJ, Sethi JM, McCool FD (2014). Diaphragm ultrasound as a predictor of successful extubation from mechanical ventilation. Thorax.

[8] Tu CS, Chang CH, Chang SC, Lee CS, Chang CT (2018). A Decision for Predicting Successful Extubation of Patients in Intensive Care Unit. Biomed Res Int 2018.

[9] Marini JJ, Wheeler AP (2006). Weaning and discontinuation of mechanical ventilation. Critical care medicine, the essentials.

[10] Epstein SK (1995). Etiology of extubation failure and the predictive value of the rapid shallow breathing index. Am J Respir Crit Care Med.

[11] Yang KL, Tobin MJ (1991). A prospective study of indexes predicting the outcome of trials of weaning from mechanical ventilation. N Engl J Med.

[12] Vallverdu I, Calaf N, Subirana M, Net A, Benito S, Mancebo J (1998). Clinical characteristics, respiratory functional parameters, and outcome of a two-hour T-piece trial in patients weaning from mechanical ventilation. Am J Respir Crit Care Med.

[13] Krieger BP, Isber J, Breitenbucher A, Throop G, Ershowsky P (1997). Serial measurements of the rapid-shallow- breathing index as a predictor of weaning outcome in elderly medical patients. Chest.

[14] Miu T, Joffe AM, Yanez ND, Khandelwal N, Dagal AH, Deem S (2014). Predictors of reintubation in critically ill patients. Resp Care.

[15] Crawford J, Otero R, Donnino M, Garcia J, Khazal R, Lenoir T (2007). Rapid shallow breathing index–a key predictor for noninvasive ventilation. Crit Care.

[16] Soleimanpour H, Taghizadieh A, Salimi R, Golzari SE, Mahmoodpoor A, Safari S (2014). Rapid shallow breathing index survey, a predictor of non-invasive ventilation necessity in patients with chronic obstructive pulmonary disease exacerbation:An analytical descriptive prospective study. Iran Red Cresc Med J.

[17] Chao DC, Scheinhorn DJ (2007). Determining the best threshold of rapid shallow breathing index in a therapist-implemented patient-specific weaning protocol. Resp Care.

[18] El Khoury MY, Panos RJ, Ying J, Almoosa KF (2010). Value of the PaO2:FiO2 ratio and Rapid Shallow Breathing Index in predicting successful extubation in hypoxemic respiratory failure. Heart &Lung. J Acute Crit Care.

[19] Seeley EJ, McAuley DF, Eisner M, Miletin M, Matthay MA, Kallet RH (2008). Predictors of mortality in acute lung injury during the era of lung-protective ventilation. Thorax.

